# Alcohol Addiction, Gut Microbiota, and Alcoholism Treatment: A Review

**DOI:** 10.3390/ijms21176413

**Published:** 2020-09-03

**Authors:** Shao-Cheng Wang, Yuan-Chuan Chen, Shaw-Ji Chen, Chun-Hung Lee, Ching-Ming Cheng

**Affiliations:** 1 Department of Forensic and Addiction Psychiatry, Jianan Psychiatric Center, Ministry of Health and Welfare, Tainan City 717, Taiwan; yuhsinliu87@gmail.com (C.-H.L.); babyming@gmail.com (C.-M.C.); 2 Department of Mental Health, Johns Hopkins Bloomberg School of Public Health, Baltimore, MD 21205, USA; 3 Gloria Operation Center, National Tsing Hua University, Hsinchu City 30013, Taiwan; yuchuan1022@gmail.com; 4 Program in Comparative Biochemistry, University of California, Berkeley, CA 94720, USA; 5 Department of Psychiatry, Taitung MacKay Memorial Hospital, Taitung City 950, Taiwan; shawjichen@gmail.com; 6 Department of Medicine, Mackay Medical College, New Taipei City 252, Taiwan; 7 Department of Informative Engineering, I-Shou University, Kaohsiung City 840, Taiwan; 8 Department of Food Nutrition, Chung Hwa University of Medical Technology, Tainan City 717, Taiwan; 9 Department of Natural Biotechnology, NanHua University, Chiayi County 622, Taiwan

**Keywords:** alcohol addiction, alcoholism medication, alcohol encephalopathy, gut microbiota, alcohol liver disease, gut–brain axis, gene editing therapy, CRISPR

## Abstract

Alcohol addiction is a leading risk factor for personal death and disability. In 2016, alcohol use caused 2.2% of female deaths and 6.8% of male deaths, and disability-adjusted life years (DALYs) were 2.3% in female and 8.9% in male. Individuals with alcohol use disorder are at high risk of anxiety, depression, impaired cognition performance, and illicit drug use and are comorbid with liver disease, such as alcoholic hepatitis and liver cirrhosis, which is a major cause of personal death and disability worldwide. Psychological interventions, such as cognitive behavior therapy and motivational interviewing, as well as medical treatments, such as disulfiram, naltrexone, acamprosate, and nalmefene, are used for the treatment of alcohol addiction in Europe and the United States. However, the effect of current interventions is limited, and the need for additional interventions is substantial. Alcohol use impairs the intestinal barrier and causes changes to the intestinal permeability as well as the gut microbiota composition. Emerging studies have tried to reveal the role of the gut–brain axis among individuals with alcohol use disorder with or without alcohol liver disease. Bacterial products penetrate the impaired intestinal barrier and cause central inflammation; changes to the gut microbiota impair enterohepatic circulation of bile acids; alcohol abuse causes shortage of vital nutrients such as thiamine. Several studies have suggested that probiotics, through either oral administration or fecal microbiota transplantation, increased intestinal levels of potentially beneficial bacteria such as bifidobacteria and lactobacilli, improving the levels of liver-associated enzymes in patients with mild alcoholic hepatitis, and demonstrating beneficial psychotropic effects on anxiety and depression. In addition to medications for alcohol addiction, gene editing therapy such as clustered regularly interspaced short palindromic repeats (CRISPRs) may be another potential research target. Alcohol dehydrogenase (ADH) and aldehyde dehydrogenase (ALDH), which are associated with ADH and ALDH genes, are major enzymes involved in alcohol metabolism, and gene editing approaches may have the potential to directly modify specific genes to treat alcoholism caused by genetic defects. Further research is needed to study the effect of the combined treatment for alcohol addiction.

## 1. Introduction

Alcohol use disorder (AUD), or what is commonly called alcohol addiction, is a leading risk factor for personal death and disability; it affects approximately 4% of the adult population [1]. In 2016, alcohol use caused 2.2% of female deaths and 6.8% of male deaths, and disability-adjusted life years (DALYs) were 2.3% in female and 8.9% in male [2]. Alcohol has significantly adverse effects on lifespan and quality of life for individuals with AUD and their families [3]. Alcohol addiction is a brain disorder involving the brain reward circuit, and individuals with AUD are at high risk of anxiety, depression, impaired cognition performance, and illicit drug use. Alcohol addiction is strongly comorbid with liver disease, such as alcoholic hepatitis and liver cirrhosis, which is a major cause of personal death and disability worldwide [4]. Alcohol addiction is a chronic illness characterized by relapse and remission [5]. According to the Diagnostic and Statistical Manual of Mental Disorders 5th edition (DSM-V), AUD is defined as a collection of brain function impairment and uncontrolled behavior, including tolerance development, withdrawal, uncontrolled increasing intake, and craving for alcohol [6]. Alcohol addiction, like other substance addiction, causes alcohol seeking and maintenance of alcohol use, involving several neurotransmitter systems such as dopamine, serotonin, opioid peptides, glutamate, and γ-aminobutyric acid (GABA) [7]. Medical treatments targeting the above neurotransmitter systems such as disulfiram (an aldehyde dehydrogenase inhibitor), naltrexone (an opioid receptor antagonist), nalmefene (an opioid receptor modulator), and acamprosate (multiple targets) are used for the treatment of alcohol addiction in Europe and the United States, as well as psychological interventions such as cognitive behavior therapy and motivational interviewing [8,9,10].

The clinical outcomes of current interventions vary, and interventions only show a small effect on improving the relapse rate. Many individuals with AUD progress to alcohol-related physical illnesses, so the need for additional interventions is substantial. Alcohol liver disease is highly associated with alcohol addiction. Alcohol use impairs the integrity of the intestinal barrier, increasing the permeability of the intestine and changing the composition of gut flora; these changes have several consequences: central inflammation due to bacterial products from the intestine, impairment of enterohepatic circulation of bile acids, and shortage of thiamine due to poor intake [11,12]. For the purpose of preventing alcohol liver disease, emerging studies try to elucidate the role of the gut–brain axis among individuals with AUD with or without alcohol liver disease. Several studies have suggested that probiotics improve the composition of gut microbiota by increasing the potentially beneficial bacteria, including bifidobacteria and lactobacilli, having benefits on both physical and mental health in patients with mild alcoholic hepatitis [13].

However, the association between alcohol addiction and the gut–liver–brain axis is unclear; fewer articles have focused on how imbalance in the gut–liver–brain axis can affect brain function of individuals with AUD, specifically cognitive functions. We will review the current literature and try to find the link between alcohol addiction and the gut–liver–brain axis. Furthermore, we will find potential treatment targets for alcohol addiction.

## 2. Alcohol Use Disorder and Cognition Impairment

AUD is operationally defined in the DSM-V as a pathologic condition manifested by 11 criteria [6]. Most individuals begin to drink alcohol for euphoric feelings or recreational use; then, tolerance develops due to the desensitization of GABA receptors and/or liver enzyme activation, leading to an uncontrolled intake. Alcohol withdrawal symptoms include anxiety, shakiness, sweating, vomiting, tachycardia, mild fever, and even more severe symptoms such as seizures, visual and auditory hallucinations, and delirium tremens [14]. Many individuals with AUD drink again and again to alleviate these intolerable withdrawal feelings [15]. The symptoms of alcohol tolerance and withdrawal, with uncontrolled intake and craving, are the core symptoms of alcohol addiction, indicating neurologic adaptation or so-called physiologic dependence.

Alcohol involves the reward system which is mediated through its effects on dopamine neurons in the mesolimbic system. The primary effects of alcohol are to inhibit N-methyl-d-aspartic acid (NMDA) receptors and to facilitate GABA receptors [16]. Individuals with AUD experience behavioral changes that indicate their cognitive function impairment, and these changes may continue even after someone has stopped drinking. Even worse, individuals with AUD are at a high risk of Wernicke encephalopathy and alcoholic Korsakoff syndrome, also called Wernicke–Korsakoff syndrome, which is caused by thiamine (vitamin B1) deficiency. The diagnostic criteria for Korsakoff syndrome include forgetting quickly, difficulty learning, and prominent amnesia, also known as alcohol-induced amnestic confabulatory. That means long-term alcohol addiction causes permanent intellectual functional impairment such as cognitive impairment and poor memory [17,18]. Consequently, individuals with AUD with intellectual impairment are unable to hold down a steady job or to maintain healthy relationships with their family.

Based on the above, tolerance and withdrawal symptoms cause individuals with AUD to continuously drink more and more alcohol; increased positive reinforcement and impaired cognitive function aggravate the intensity of the alcohol addiction of individuals with AUD. Thus, effective medical interventions are needed to prevent the cognitive deterioration of individuals with AUD.

## 3. Thiamine Deficiency and Alcohol Encephalopathy (Wernicke–Korsakoff Syndrome)

**Figure 1:**
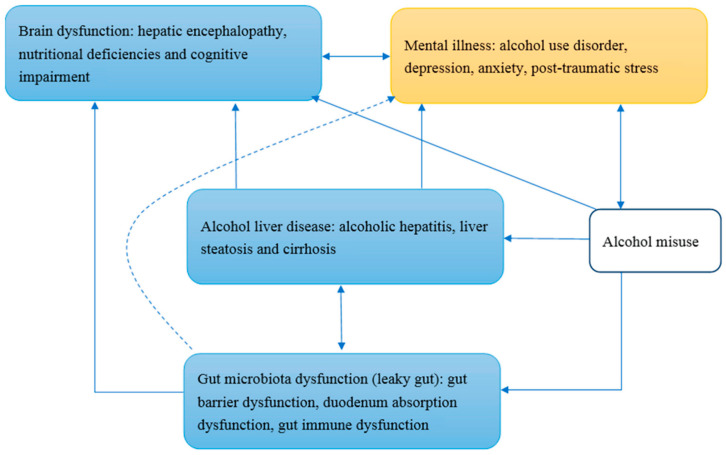
The conceptual framework of alcohol use disorder and the gut–brain–liver axis.

Wernicke–Korsakoff syndrome includes Wernicke encephalopathy and alcoholic Korsakoff syndrome; Wernicke encephalopathy is characterized by acute neurologic defects such as ophthalmoplegia, confusion, and ataxia, and Korsakoff syndrome is a chronic neurologic sequela of long-term thiamine deficiency. Individuals with Korsakoff syndrome often show spontaneous confabulations, which are produced by a deficit in source memory; to fill in for the source memory they cannot access, they confabulate with irrelevant or inappropriate old memory. In addition, spontaneous confabulations are associated with executive dysfunction including inability to inhibit incorrect memories and/or to shift their attention away [19]. Individuals with Wernicke–Korsakoff syndrome often have atrophy/infarction of specific brain regions including the thalamus, basal forebrain, raphe nuclei, cerebellum, and especially mammillary bodies [20,21]. Thiamine deficiency is related to absorption impairment in the duodenum due to alcohol consumption, malnutrition from poor diet, and impaired storage in the liver [22] (Figure 1). Consequently, Kreb’s Cycle impairment due to thiamine deficiency results in inadequate production of energy for the brain cell functioning; brain neurons die when the high amount of energy required is not supplied [23].

Due to poor absorption and/or other gastrointestinal illness, individuals with AUD with Wernicke encephalopathy are given high doses of thiamine intravenously or intramuscularly to prevent Korsakoff syndrome or reduce its severity. Thiamine administration can reduce the progression of neurologic deficits caused by Wernicke–Korsakoff syndrome in around 21 to 25% of patients [12,24]. In addition to the direct effect of alcohol on brain function, alcohol can damage the intestinal wall and cause gut microbiota dysfunction, inducing malabsorption of thiamine, and as we mentioned above, the deficiency of thiamine plays an important role on the brain dysfunction.

Alcohol misuse can induce alcohol liver disease, gut microbiota dysfunction, and also partly brain dysfunction. Alcohol liver disease and gut microbiota dysfunction may have reciprocal interaction through enterohepatic circulation. Alcohol liver disease induces brain dysfunction as well as mental illnesses. Gut microbiota dysfunction may induce brain dysfunction through systemic inflammation and malnutrition, including thiamine deficiency. The dotted line in Figure 1 shows that the association between gut microbiota dysfunction and mental illnesses is unclear. Mental illnesses interact with brain dysfunction, as well as alcohol misuse; for example, individuals with depression and/or post-traumatic stress disorder are at high risk of alcohol misuse. Alcohol is a central nervous system depressant, and alcohol misuse directly induces brain dysfunction. The blue squares in Figure 1 are components of the gut–brain–liver axis, and the yellow square presents mental illnesses such as alcohol use disorder.

## 4. Alcohol Addiction and the Gut–Brain–Liver Axis

As we stated above, alcohol addiction is associated with absorption impairment in the duodenum, leading to malnutrition and impaired liver storage of thiamine. Alcohol liver disease is one of the most common preventable diseases in the world, including asymptomatic liver steatosis, fibrosis, cirrhosis, and alcoholic hepatitis [25]. Among patients with AUDs, alcohol liver disease is the most common cause of death [26,27].

In the past decade, the association between the gut, or particularly the gut microbiota, and alcohol liver disease has raised researchers’ attention. Among individuals with alcohol addiction, alcohol consumption breaks down the gut barrier function, also called leaky gut [28,29]. The intestinal barrier is composed of enterocytes, goblet cells and antimicrobial substances affecting the intestinal microbiome within a mucus layer, and numerous immune cells in the lamina propria [30]. We do not clearly understand how alcohol, or its metabolite acetaldehyde, induces leaky gut. However, intestinal dysbiosis, immune system activation, and inflammation may play essential roles.

Intestinal dysbiosis is the alteration of the gut microbiota, which is characterized by intestinal bacterial taxa changes such as decreased levels of anti-inflammatory bacteria, including *Faecalibacterium prausnitzii* and *Bifidobacterium*, and increased abundance of Proteobacteria [31,32]. Animal studies support that improvement in intestinal barrier integrity can ameliorate alcohol-induced liver damage [33,34,35]. This indicates that gut microbiome modulation treatment such as probiotics may have beneficial effects on AUDs. Continuous alcohol abuse can change the fecal pH, promoting overgrowth of pathogens, and is also associated with alterations of the gut microbiota functionality by changing specific metabolite secretions involved in gut barrier dysfunction [36,37]. Individuals with AUD often show elevated levels of plasma cytokines such as TNFα, interleukin 10, and CRP, suggesting chronic, low-grade, systemic inflammation [38]. In the past decade, several studies have revealed that there is an association between systemic inflammation and psychiatric disorders including depression and autism [39,40]. However, the mechanism of interaction between systemic inflammation and psychiatric disorders such as alcohol addiction, depression, and autism are not fully understood, and gut microbiota may be a good research target. A possible mechanism of systemic inflammation and alcohol addiction is that the intestinal bacterial products activate peripheral blood mononuclear cells, inducing cytokines entering the bloodstream, causing low-grade, systemic inflammation among individuals with alcohol dependence [38].

Alcohol addiction is highly associated with other psychiatric illnesses, including major depressive disorder, bipolar disorders, as well as anxiety disorders [41]. Mood disorders such as major depressive disorders often precede the onset of alcohol addiction; for example, individuals use alcohol to cope with low mood. The severity of alcohol addiction is correlated with the intensity of their craving, cognitive dysfunction, anxiety, and depressive symptoms. As we mentioned above, systemic inflammation may play an important role in the development of alcohol addiction; gut barrier dysfunction and inflammation in both the gut and liver may contribute to peripheral inflammation and cause brain inflammation [38], inducing inflammation of brain cells such as microglia or astrocytes [42]. The sickness behavior theory may link systemic inflammation to both alcohol addiction and mood disorders [43]. This theory supports that peripheral inflammation, such as leaky gut, activates the immune system and produces cytokines that can reach the brain, causing fever, fatigue, lassitude, inability to concentrate, and withdrawal from social interactions; when the above behaviors persist, depressive symptoms may develop [44]. Though studies support alcohol addiction or craving is associated with systemic inflammation, the causality is still unclear. Nevertheless, the combination of alcohol craving and negative emotions such as anxiety and depression is highly associated with drug-seeking behaviors and relapse, indicating that reducing systemic inflammation may improve psychological well-being and prevent relapse [45].

## 5. Current and Potential Medical Treatment for Alcohol Addiction

Alcohol addiction involves various neurotransmitters and their receptors in the brain, including dopamine, serotonin, opioid peptides, glutamate, and GABA [4]. Current pharmacological treatment for alcohol addiction targets the above neurotransmitter systems. Three medications for alcohol addiction are approved in the United States by the Food and Drug Administration: disulfiram, acamprosate, and naltrexone. Nalmefene is approved in Europe by the European Medicines Agency and is recommended by the National Institute for Health and Care Excellence in the United Kingdom. The details of the above medications are described below.

Disulfiram is an antabuse, also known as an aversive agent. Disulfiram inhibits aldehyde dehydrogenase (ALDH) and prevents the elimination of acetaldehyde, which causes hangover symptoms among individuals with AUD. Those who take disulfiram will have an immediate, extremely uncomfortable hangover when they drink alcohol [46]. Acamprosate acts on GABA and the glutamate system; the possible mechanism is that acamprosate can reduce the intensity of alcohol withdrawal, thus reducing the risk of relapse [8]. Naltrexone is an opioid antagonist, which blocks the effects of endorphins and opioids. μ opioid receptor and β-endorphin induce a euphoric feeling among individuals with AUD, and evidence supports that blocking the euphoric feeling can reduce risk of relapse and excessive drinking [8]. In addition, long-acting injectable naltrexone can alleviate cravings and decrease the risk of opioid overdose [47]. Nalmefene is a μ and δ opioid receptor antagonist, and a κ opioid receptor partial agonist. It appears to work in a similar manner as naltrexone, but the mechanism is under investigation. Several other drugs for alcohol-related illnesses are also described below, but the mechanisms are still under investigation. Benzodiazepines are used to treat acute alcohol withdrawal and work on the similar GABA receptor as ethanol. Baclofen, a GABA agonist, is also used to treat AUDs. The mechanism and effect of calcium carbimide are similar to disulfiram [48]. Topiramate, used to treat epilepsy and prevent migraines, is supported to treat alcohol-related illnesses, and gabapentin, also used to treat epilepsy, is supported to treat alcohol withdrawal [49,50].

As previously mentioned, thiamine administration intravenously or intramuscularly is used among individuals with Wernicke encephalopathy to prevent Korsakoff syndrome or reduce its severity. Another important issue, alcohol liver disease, has raised researchers’ attention recently. On the basis of growing evidence that the interaction between gut microbiota and intestinal inflammation may play an important role in the development of alcohol liver disease, several pioneer studies have investigated the effect of gut microbiota modification and showed the potential to reduce liver enzyme levels to improve systemic inflammation and endotoxemia [30,32,51]. Though recent studies support that systemic inflammation, as well as immunity responses, is associated with psychological well-being, current studies related to gut microbiota modification have mainly focused on alcohol liver disease. We suppose that gut microbiota modification is not only beneficial to alcohol liver disease but also alcohol addiction, through reducing the intensity of gut inflammation and improving absorption of nutrients such as thiamine; however, studies related to the association between gut microbiota modification and alcohol addiction are still scarce.

Transcription activator-like effectors nucleases (TALENs) and clustered regularly interspaced short palindromic repeats (CRISPRs) are two successful approaches applied in gene-editing of organisms [52]. Currently, CRISPRs are the most extensive approach because they have more advantages over the other technologies, including no species limitation, simplicity, efficiency, precision, and economy.

CRISPR/Cas9 was originally found as a bacterial adaptive immune system which acts against invading plasmids or viruses [53]. CRISPRs are loci that contain multiple, short, direct repeats of DNA sequences in bacteria. Each repeat contains a series of base pairs followed by about 30 base pairs known as “spacer DNA”. Spacers are short segments of DNA from a virus and serve as a ‘memory’ of past exposures. The host bacteria can recognize foreign DNA by complementation with the stored short spacer sequence once they encounter the same virus again. In the CRISPRs/Cas9 system, a hybrid of crRNA transcribed from CRISPR loci and transactivating crRNA (tracrRNA) acts as a single-guide RNA (sgRNA) to initiate destruction of the invading DNA/RNA by Cas endonucleases [54,55].

A complicated mixture which consists of genetic and environmental factors is associated with the risk of developing alcoholism [56]. The genes that affect alcohol metabolism may be indicated by a family history of alcoholism. Alcohol dehydrogenase (ADH) and ALDH are major enzymes involved in alcohol metabolism; thus, noncoding variants in ADH and ALDH genes may influence alcohol metabolism. Both enzymes exist in several forms encoded by different ADH and ALDH genes, among them, ADH1B and ALDH2 are the two known genes that are strongly related to risk for alcoholism [57]. Genetic variations in different individuals affect the risk of developing alcohol dependence. For example, the allele ADH1 B*3 induces a more rapid metabolism of alcohol, and thereby individuals with this allele have a lower risk of developing alcoholism [58]. These findings hint at the possibility of directly modifying specific genes (e.g., ADH1B and ALDH2) using gene-editing approaches (e.g., CRISPR/Cas9) to treat alcoholism caused by genetic defects. We summarized the current and potential medical treatments for alcohol-related illness and listed indications, approval authorities, and the possible mechanisms of action in Table 1.

## 6. Conclusions

In 2016, alcohol use caused numerous deaths, reduced DALYs worldwide, and was the seventh leading risk factor for disease burden, in particular among the working-age population. In addition, the other two leading causes of death in this particular age group were road injuries and self-harm, which are also highly associated with alcohol consumption [2]. Alcohol addiction among this working-age population may change the course of their lifetime; craving for alcohol and non-stop drinking can ruin an individual’s employment opportunities and can even lead to severe physical illness such as liver disease, cardiovascular disease, and different types of cancer. Moreover, alcohol addiction causes poor intake and impairs an individual’s cognitive function.

Medications for alcohol-related illnesses are proven to be somewhat effective, with different mechanisms for different medications. Disulfiram inhibits aldehyde dehydrogenase and causes hangover symptoms among individuals with AUD when they drink. Acamprosate reduces the intensity of alcohol withdrawal, as well as the risk of relapse. Naltrexone blocks the euphoric effects of endorphins and opioids, reducing risk of relapse and excessive drinking. Nalmefene works in a similar manner as naltrexone, but the mechanism is still unclear. Targeting on the GABA system, benzodiazepines are used to treat acute alcohol withdrawal, and Baclofen is used to treat AUDs. Several medications for epilepsy, including Topiramate, are used to treat alcohol-related illnesses, and gabapentin is used to treat alcohol withdrawal.

Alcohol addiction is associated with duodenum absorption dysfunction, malnutrition, and impaired liver function. Alcohol liver disease, including asymptomatic liver steatosis, fibrosis, cirrhosis, and alcoholic hepatitis, is the most common cause of death and can be prevented by alcohol abstinence. Recently, the association between the gut microbiota, alcohol liver disease, and alcohol addiction has raised researchers’ attention. Systemic inflammation caused by leaky gut and alcoholic hepatitis may play an important role in the development of psychiatric disorders such as depression, anxiety, and cognitive impairment among individuals with AUD. In addiction to alcohol liver disease, Wernicke–Korsakoff syndrome, characterized by neurologic deficit and memory impairment, is caused by long-term alcohol consumption and deficiency of thiamine. Impaired cognitive function may be associated with the development of alcohol addiction and is also a marker of addiction severity.

Thiamine administration is used among individuals with AUD to treat Wernicke–Korsakoff syndrome, which is then replaced by an oral form thiamine after the individuals have improved. As we previously mentioned, several studies have investigated the effects of gut microbiota modifications and showed the potential benefit to reduce liver enzyme levels among individuals with AUD. Based on the above, gut microbiota modification can improve liver function, thiamine supplementation can somewhat reverse the impaired cognitive function due to thiamine deficiency, and approved pharmacological medications for alcohol addiction may modify several neurotransmitters to reduce alcohol craving or consumption. Combining treatments such as gut microbiota modification and thiamine supplementation with the current pharmacological treatments for alcohol addiction may have potential benefits. 

Another potential treatment, gene editing approaches, has been used for genetic diseases such as α1-antitrypsin deficiency, β-thalassemia, sickle cell anemia, muscular dystrophy, Leber congenital amaurosis, and cystic fibrosis [52,59]. However, most gene therapies based on these techniques are still under research because there are many challenges including technology limitations, safety concerns, and ethical/moral issues [52]. Although gene editing approaches might expedite the progression of gene therapy for the treatment of alcoholism, the associated challenges must be overcome to maximize the potential of gene therapy to translate to the clinics and improve healthcare outcomes. For clinical application, identification of candidate genetic variants that regulate responses to these novel medications which treat alcoholism is needed. Fortunately, the growing availability of genetic health record data has enabled the establishment of large-scale and electronic data banks, and these banks are advantageous to offer important opportunities to develop this area of research for the treatment of alcoholism.

The aim of alcohol addiction treatment is to stabilize patients’ physical and psychiatric problems; medications including gut microbiota modification, thiamine supplementation, and approved pharmacological medications may have the potential to modify the associations to rewarding stimuli from alcohol to daily life events and change the behavior patterns of individuals with AUD. Gene editing therapy may be another potential treatment for alcohol addiction, but this is currently under investigation. Further research is needed to study the effects of combined treatment for alcohol addiction.

## Figures and Tables

**Table 1:** Current and potential treatments for alcohol-related illness.

Treatments	Indication/Authorities Approval	Mechanisms of Action
**current pharmacological treatments**		
**disulfiram (antabuse)**	Use ONLY as adjunct to supportive and psychotherapeutic treatment, in motivated patients /FDA approved	Aversive agent, ALDH inhibitor (blocks the metabolism of alcohol’s primary metabolite acetaldehyde)
**naltrexone (nalorex, depade, revia vivitrol)**	Treatment in patients who have been able to abstain from alcohol, decrease the desire, the amount and the frequency of drinking before treatment initiation /FDA approved	μ-opioid receptor antagonist, blocks β-endorphin release induced by alcohol
**nalmefene (selincro)**	reduce alcohol consumption in combination with psychological support for people who drink heavily /EMA approved	μ and δ-opioid receptor antagonist; κ-opioid receptor partial agonist
**acamprosate (campral, aotal)**	Indicated for maintenance of alcohol abstinence in patients who are abstinent at treatment initiation /FDA approved	Acts on GABA and glutamate neurotransmitter systems; still under investigation
**gabapentin (neurontin)**	Reduce symptoms of alcohol withdrawal, alcohol dependence, and craving /Still under investigation	GABA inhibitor and calcium channel blocker
**baclofen (lioresal)**	Still under investigation /Temporary recommendation issued by the French drug agency ANSM	GABA receptor agonist
**current diet supplements**		
**thiamine**	Treat and prevent thiamine deficiency and disorders that result from it, such as Wernicke encephalopathy /Diet supplement	Vitamin B1 supplement
**probiotics (*Faecalibacterium prausnitzii*, *Bifidobacterium*, and others)**	Improve intestinal barrier integrity and ameliorates alcohol-induced liver damage /Diet supplement	Alteration of the gut microbiota by changing secretion of specific metabolites involved in gut barrier dysfunction, might improve vitamin B1 absorption; still under investigation
**potential treatments**		
**gene-editing therapy (talens and crisprs)**	Still under investigation	Might target the gene related to ADH and ALDH, the major enzymes involved in alcohol metabolism

Abbreviations: ADH, alcohol dehydrogenase; ALDH, aldehyde dehydrogenase; GABA: γ-aminobutyric acid; EMA, European Medicines Agency; FDA, Food and Drug Administration; ANSM, Agence nationale de sécurité du medicament; TALENs, transcription activator-like effectors nucleases; CRISPRs, clustered regularly interspaced short palindromic repeats.
